# Leptomeningeal carcinomatosis and brain metastases in gastroesophageal carcinoma: a real-world analysis of clinical and pathologic characteristics and outcomes

**DOI:** 10.1007/s11060-024-04576-8

**Published:** 2024-02-19

**Authors:** Thais Baccili Cury Megid, Zeynep Baskurt, Lucy X. Ma, Carly C. Barron, Abdul Farooq, Marie Phillipe Saltiel, Xin Wang, Yvonne Bach, Hiroko Ayoama, Raymond W. Jang, Eric Chen, Patrick Veit-Haibach, Ben Wang, Sangeetha Kalimuthu, James Cotton, Rebecca Wong, Aruz Mesci, Elena Elimova

**Affiliations:** 1 Medical Oncology and Hematology at Princess Margaret Cancer Centre, Toronto, Canada; 2 Department of Biostatistics, Princess Margaret Cancer Centre, University Health Network, Toronto, Canada; 3 Department of Medical Oncology and Hematology, University of Toronto, Toronto, Canada; 4 Toronto Joint Department Medical Imaging and University Health Network, Sinai Health System, University Medical Imaging Toronto, Women’s College Hospital, Toronto, Canada; 5 Department of Pathology, Princess Margaret Cancer Centre, Toronto, Canada; 6 Department of Radiation Oncology, Princess Margaret Cancer Centre, Toronto, Canada

**Keywords:** Brain metastasis, Leptomeningeal carcinomatosis, Gastric cancer, Esophageal cancer, HER2

## Abstract

**Background:**

Brain metastasis (BrM) and Leptomeningeal Carcinomatosis (LMC) are uncommon complications in gastroesophageal carcinoma (GEC) patients. These patients have a poor prognosis and are challenging to treat. We described the clinicopathologic features and outcomes in the largest cohort of Central Nervous System (CNS) metastasis in GEC patients.

**Methods:**

single-center retrospective study of GEC treated from 2007 to 2021. Clinicopathologic characteristics and treatment modalities were reviewed. Survival was calculated from the date of CNS diagnosis until date of death/last follow-up using the Kaplan-Meier method. A multivariable Cox proportional hazards regression model was used.

**Results:**

Of 3283 GEC patients, 100 (3.04%) were diagnosed with BrM and 20 with LMC (0.61%). Patients with known human epidermal growth factor receptor 2 (HER2) status (*N* = 48), 60% were HER2 positive (defined as IHC 3 + or IHC 2+/FISH+). Among LMC patients most were signet-ring subtype (85%), and only 15% (2/13) were HER2 positive. Median survival was 0.7; 3.8; and 7.7 months in BrM patients treated with best supportive care, radiation, and surgery, respectively (*p* < 0.001). In LMC, median survival was 0.7 month in patients who had best supportive care (7/19) and 2.8 months for those who had whole brain radiation therapy (*p* = 0.015). Multivariate analysis showed worse outcomes in ECOG ≥ 2 (*p* = 0.002), number of BrM ≥ 4 (*p* < 0.001) and number of metastatic sites (*p* = 0.009).

**Conclusion:**

HER2 expression were enriched in patients with BrM, while it is uncommon in LMC. Patients treated with surgery followed by radiation had an improved OS in BrM and WBRT benefited patients with LMC.

## Introduction

Gastric adenocarcinoma is the fifth most common malignancy and the fourth most frequent cause of cancer-associated death worldwide, with approximately 1,100,000 new incident cases and about 800,000 related mortalities estimated in 2020 [1–2]. Regarding esophageal cancer, there were approximately 604,100 newly reported cases and 544,100 fatalities. Predominantly, 85% of the cases (512,500 cases) were squamous cell carcinomas (SCCs), while adenocarcinoma accounted for 14% of the cases (85,700 cases) [1].

Brain metastasis (BrM) and leptomeningeal carcinomatosis (LMC) are rare complications associated with gastroesophageal carcinoma (GEC). The occurrence of BrM ranges from 0.7 to 6.6% [3–9], while the incidence of LMC varies between 0.16% and 0.19% [10]. The complications are associated with neurologic morbidity and poor prognosis and quality of life [11], with median survival historically reported to be in the range of 2 to 6 months after its diagnosis [3–8].

Clinical risk factors for BrM development are poorly understood, although associations have been found with higher lymph node (N) stage and the presence of other metastases (particularly liver, lung, and bone) [5]. Several studies have noted the enrichment of human epidermal growth factor receptor 2 (HER2) overexpression among patients with gastroesophageal cancer and brain metastases; however, it is unclear if this is a risk factor in patients with leptomeningeal carcinomatosis [12–14].

Treatment of brain metastasis and leptomeningeal carcinomatosis is challenging because most systemic chemotherapeutics in use have limited permeability across the blood brain barrier [15–16]. Therefore, treatment of brain metastasis relies on surgical resection and/or radiotherapy as treatment modalities and leptomeningeal carcinomatosis, on whole brain radiation therapy and, in selected cases, intrathecal chemotherapy [8–10].

Given the limited data available regarding clinical features, prognostication, and treatment of CNS metastasis from gastroesophageal carcinoma, the objective of our study was to characterize the clinical, molecular features and outcomes of patients in this comprehensive cohort population investigation.

## Methods

### Study population and data collection

This study is a retrospective analysis that included adult (age ≥ 18 years) patients treated at the Princess Margaret Cancer Center (PMCC) between 2007 and 2021 with a confirmed diagnosis of GEC, from which we identified patients with disease metastatic to the brain or leptomeninges. The data were collected from the electronic medical record into the Gastroesophageal Database using Research Electronic Data Capture (REDCap) and the study was approved by the University Health Network Research Ethics Board (REB) - CAPCR ID (REB ID 20-5884). - and adhered to the data confidentiality and privacy policy of the International Credential Evaluation Service.

For each included patient, the clinical staging was determined using the American Joint Committee on Cancer (AJCC 6) staging manual, given the retrospective nature of this cohort [17]. This study included adenocarcinomas of the distal esophagus, esophagogastric junction, and proximal stomach, categorized respectively as types I, II, and III using the Siewert classification [18]. In addition, pure squamous cell carcinomas were also included, while neuroendocrine, and undifferentiated carcinomas were excluded. Two patients were found concomitantly with both brain metastasis and leptomeningeal carcinomatosis, and they were included in the LMC group analysis.

Patient characteristics, including age at diagnosis, sex, ethnicity, and Eastern Cooperative Oncology Group (ECOG) Performance Status (PS), were recorded. Tumor characteristics, including date of diagnosis, number of brain metastases, number and location(s) of extracranial metastases, clinical staging, and HER2 status, were also noted.

All patients diagnosed with brain metastasis underwent neurological imaging as part of their evaluation for neurological symptoms and confirmation of CNS metastasis. In our study, the diagnosis of leptomeningeal carcinomatosis was established by utilizing neuroimaging findings and/or cerebrospinal fluid analysis. Neuroimaging features included linear and/or nodular enhancement of the leptomeninges, cranial nerves, and spinal nerve roots. In cases where clinical symptoms suggested leptomeningeal carcinomatosis, but neuroimaging results were negative, patients underwent a lumbar puncture. Cerebrospinal fluid (CSF) analysis confirmation revealed distinctive abnormalities, such as elevated protein levels, an increased count of nucleated cells, decreased glucose, and notably, the identification of neoplastic cells.

### Outcomes

The primary objective of this study was to delineate clinicopathologic features in individuals diagnosed with CNS metastasis from GEC. Furthermore, the study aimed to accomplish various secondary goals. These included examining treatment approaches and their correlation with overall survival (OS); assessing the prevalence of CNS metastases in a substantial patient cohort; analyzing prognostic factors; and evaluating the time to develop CNS metastases; describing the most common symptoms experienced by patients.

### Statistical analysis

Patient characteristics were summarized using descriptive statistics. For categorical and discrete variables, frequency tables were produced. OS was defined as the time from diagnosis of brain metastasis or leptomeningeal carcinomatosis to death from any cause. The Kaplan-Meier method was used for time-to-event analyses. The Cox proportional hazards regression model assessed the association between characteristics and OS. The included variables were age, number of brain metastases, number of extracranial metastatic sites, treatment modality, and ECOG PS. Patients without documented evidence of an event were censored at the date of the last follow-up. Time to develop CNS metastases was calculated from the date of GEC diagnosis to the date of CNS metastases. Hazard ratios (HR) and their corresponding 95% confidence intervals (CI) were computed to assess the magnitude and precision of these associations. A significance level of 5% (*p* < 0.05) was employed to determine statistical significance. The significance difference between HER2 status in leptomeningeal and brain metastasis patients was determined using Fisher’s Exact Tests or Wilcoxon rank-sum test.

## Results

### CNS metastasis incidence and patient’s characteristics

**Fig. 1:**
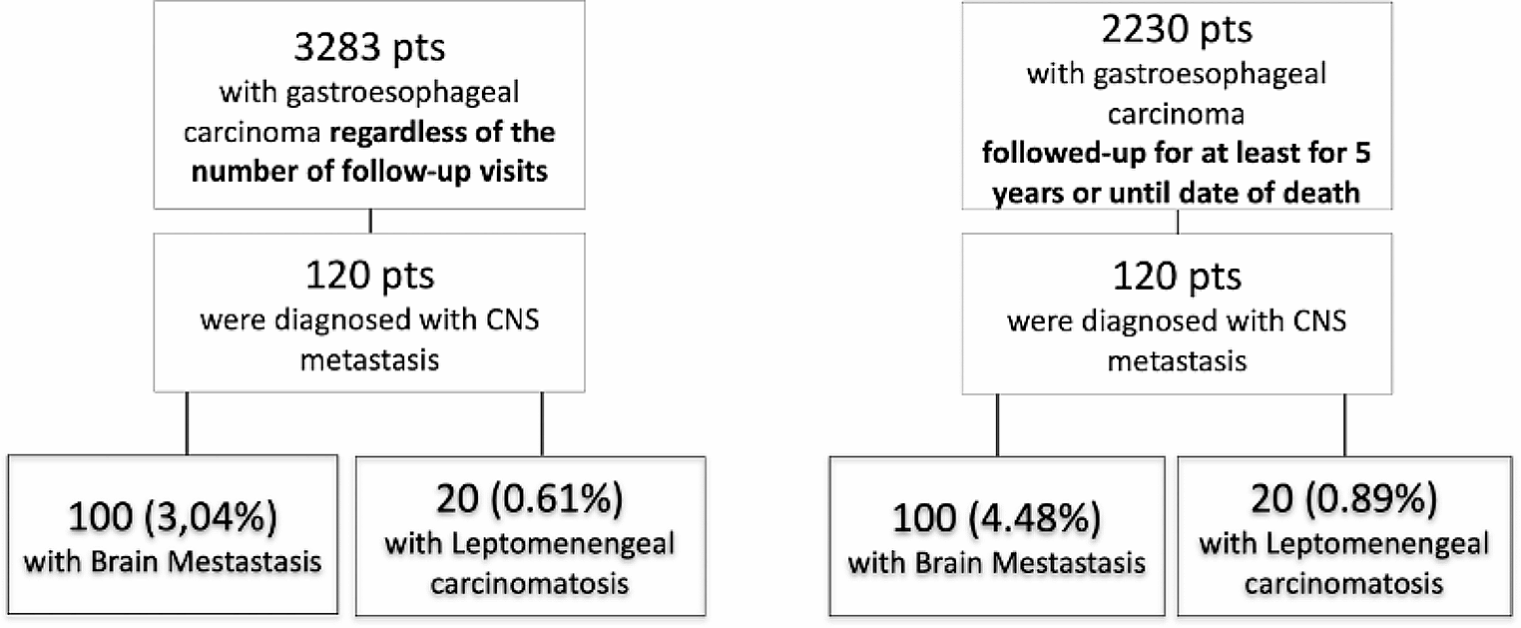
Study flow chart. presents two distinct study flow cohorts of the same population, elucidating the prevalence of CNS metastases among patients diagnosed with GEC. The initial cohort encompasses all 3,283 patients, where 100 (3.04%) were found with BrM and 20 (0.61%) with LMC, regardless of if they were treated or followed up in our institution or not. It’s noteworthy that patients decided to received treatment in other centers, potentially impacting the accuracy of reported CNS metastasis frequencies. In the second cohort, comprising 2,230 patients actively monitored until death or achieving a disease-free survival of at least 5 years, the recalculated frequencies for BrM and LMC are 4.48% and 0.89%, respectively. This recalibration providing a more accurate representation of incidence within this specific patient group

**Fig. 2:**
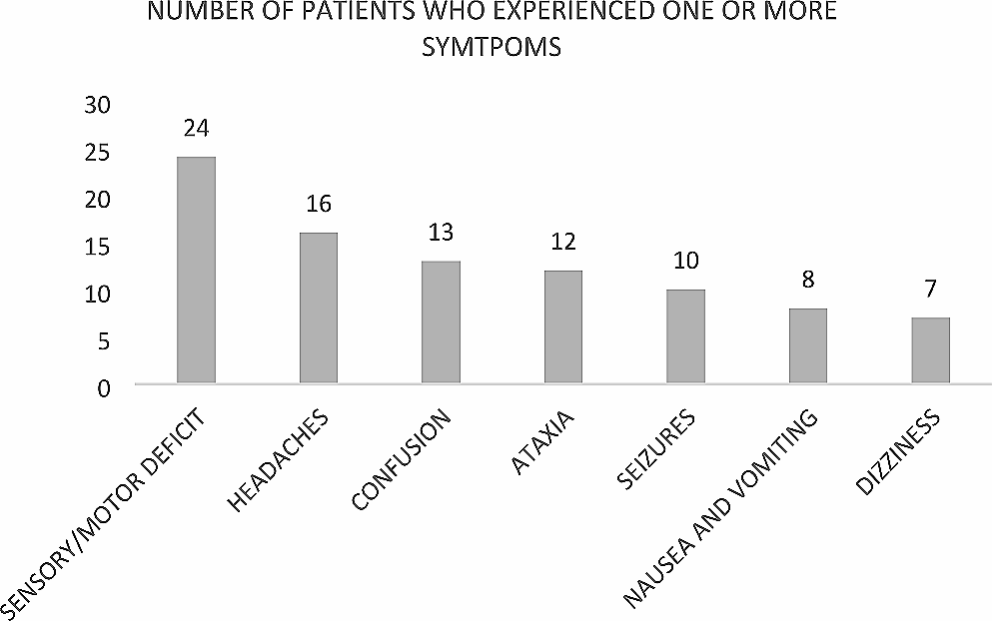
Symptoms experienced by the patients at the time at brain metastases diagnosis

Of 3283 patients diagnosed with GEC, a total of 120 (3.65%) patients were identified in our database with CNS metastases (Fig. 2). Of these, 100 (3.04%) patients were diagnosed with BrM and 20 (0.61%) were found with LMC (Fig. 2). Some patients in the database may have had only 1 or 2 initial consultations and were not followed up. Their eventual development of brain metastases may not be recorded. To address a possible more accurate incidence, we selected in our cohort patients who were actively followed until death or achieved a disease-free survival of at least 5 years. Within this refined cohort (2230 patients), the frequency of BrM and LMC was determined to be 4.48% and 0.89%, respectively (Fig. 1).

Among the patients with BrM, the median age was 64.4 years. Most patients were non-asian (*n* = 93, 93%), and 63 patients (63%) were either current or former smokers. In terms of the initial gastroesophageal staging, 65% received a de novo stage IV diagnosis, while 35% initially had stage I-III diagnoses, subsequently experiencing recurrence, and progressing to metastatic disease. Concerning the presentation of CNS metastasis, 28% presented with de novo brain metastasis, and 72% developed brain metastasis during their disease course. Adenocarcinoma histology was observed in 86 patients (86%), while 14 patients (14%) had squamous cell carcinoma (SCC). Additionally, only 19 patients (20%) had an ECOG PS score of 2 or higher at the time of BrM diagnosis (Table 1).

**Table 1:** Baseline characteristics of patients with CSN metastases from gastroesophageal carcinoma (*N* = 120)

Variable	Brain Metastases Patients, No. (%) (*n* = 100)	Leptomeningeal Carcinomatosis Patients, No. (%) (*n* = 20)	*p*-value	Statistic Test
**Age**			**0.002**	Wilcoxon Rank Sum
Mean	62.2 (10.9)	53.6 (11.2)		
Median (min, max)	64.4 (29.2, 87.8)	52.7 (30.3, 73.6)		
**Race**			0.37	Fisher Exact
Non-Asian	93 (93)	17 (85)		
Asian	7 (7)	3 (15)		
**Smoking**			0.71	Fisher Exact
Never Smokers	28 (28)	8 (40)		
Former Smokers	49 (49)	9 (45)		
Currently Smokers	14 (14)	3 (15)		
**Stage at Diagnosis of GEC**			0.30	Fisher Exact
I-III	35 (35)	4 (20)		
IV	65 (65)	16 (80)		
**Stage at Diagnosis of CNS metastasis**			**0.004**	Fisher Exact
IV	72(72)	20 (100)		
IV with denovo CNS metastasis	28 (28)	0 (0)		
**Tumor Histology**			0.12	Fisher Exact
Adenocarcinoma	86 (86)	20 (100)		
Squamous Cell Carcinoma	14 (14)	0 (0)		
**Treatment Modality**			N/A	
Surgery Followed by Radiation	25 (25)	NA		
Radiation Only (SRS/WBRT)	63 (63)	12 (65) *		
SRS	13 (21)	N/A		
WBRT	45 (71)			
*Unknown*	5 (8)			
Palliative Care Alone/Best supportive care	12 (12)	7 (35)		
**ECOG (CNS metastasis)**			0.76	Fisher Exact
0–1	80 (80)	17 (85)		
≥2	20 (20)	3 (15)		

Abbreviations: ECOG – Eastern Cooperative Oncology Group (ECOG) SRS - stereotactic radiosurgery, WBRT – Whole Brain Radiation Therapy, NA not Applicable

In the population diagnosed with LMC, the median age was 53.6 years. Out of the total, 17 individuals (85%) were non-asian, and 12 individuals (60%) were either smokers or former smokers. Additionally, 16 individuals (80%) were diagnosed with stage IV disease at the time of GEC diagnosis. Notably, there were no patients with de novo leptomeningeal carcinomatosis; all cases of leptomeningeal carcinomatosis developed at some point in their course. All 20 individuals (100%) had adenocarcinoma histology, and 3 individuals (15%) had an ECOG Performance Status of 2 or higher at the time of the diagnosis of LMC as shown in Table 1.

### Diagnostic methods

Among those with leptomeningeal carcinomatosis in our study, 16 patients (80%) received a diagnosis solely through brain MRI without requiring further confirmation through a lumbar puncture. However, for the remaining 4 patients (20%), the diagnosis was established through a lumbar puncture. None of these patients were treated with intrathecal chemotherapy.

### Symptoms at CNS presentation

**Fig. 3:**
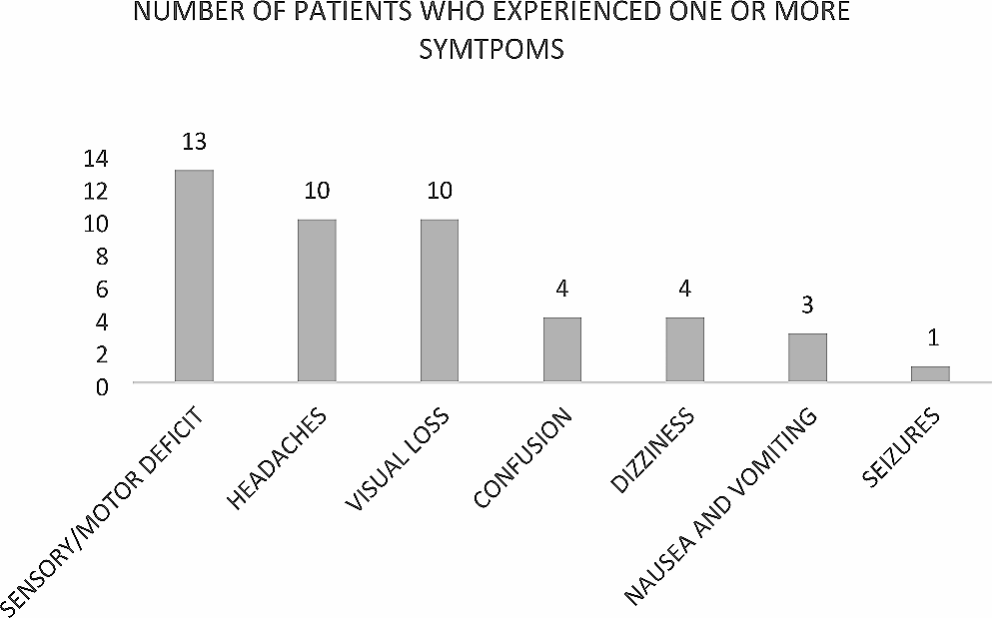
Symptoms experienced by the patients at the time of leptomeningeal carcinomatosis diagnosis

All patients in this study presented neurological symptoms at the time of CNS involvement. Patients could have experienced more than one symptom at the time of CNS presentation. Among patients with brain metastasis, the most prevalent symptoms were sensory, or motor neurological deficits (24 patients) followed by headaches (16 patients), as described in Fig. 2. Sensory or motor neurological deficits (13 patients), headaches (10 patients) and visual loss (10 patients) were the primary symptoms observed in patients with leptomeningeal carcinomatosis. (Fig. 3).

### Histological and molecular characteristic

The molecular and histological characteristics were derived from the primary tumor. It’s important to note that not all patients who underwent lumbar puncture or brain metastasis resection had their tumors retested for molecular characteristics, therefore, histological, and molecular features were not compared between the primary and corresponding metastatic tissue sample.

HER2-positive disease was defined as a score of 3 on immunohistochemical (IHC) analysis or a score of 2 + on IHC analysis with a positive result from subsequent fluorescent in situ hybridization testing. In patients where the HER2 status were known (41 patients), 61% (25 patients) were found to be HER2-positive. In the leptomeningeal carcinomatosis population with known HER2 status (13 patients), only 2 (15%) patients were HER2-positive (*p*-value 0.009) (Table 2). In terms of histology classification based on the Lauren system [19], among LMC patients, 12 (86%) had the diffuse type and 16% had the Intestinal type and no SCC were found in this population (*p*-value < 0.001) (Table 2).

**Table 2:** Histology characteristics*

Variable	Brain Metastases Patients, No (*n* = 100), %	Leptomeningeal Carcinomatosis Patients (*n* = 20), %	*p*-value	Statistic Test
**HER-2 status ** ***(excluded SCC histology)***				
Positive	25 (61)	2 (15)		
Negative	16 (39)	11 (85)		
			*0.009*	*Fisher’s exact test*
Unknown	44	7		
**Histology Classification (Lauren)**			*< 0.001.*	*Fisher’s exact test*
Diffuse Type	7 (23)	12 (86)		
Intestinal	9 (30)	2 (14)		
Indeterminate / SCC	14 (47)	0 (0)		
Unknown	70	6		

*Histology tissue from primary tumor or metastasis biopsy

### Treatment modalities

In relation to treatment approaches, a total of 25 (25%) patients with brain metastases underwent a combination of surgery followed by radiation therapy. Among the BrM patients, 63 (63%) received radiation therapy alone, which could either be stereotactic radiosurgery (SRS) (13 patients) or whole brain radiation therapy (WBRT) (45 patients).

Five patients (8%) underwent radiation treatment outside our institution, and there is no record documenting the specific modality of radiation treatment administered. Additionally, 12 (12%) BrM patients did not undergo any CNS or systemic treatment (best support of care).

For patients diagnosed with leptomeningeal carcinomatosis, 12 (65%) individuals received WBRT as their treatment modality. On the other hand, 7 (35%) patients had no specific treatment modality (Table 1).

The only two patients with HER2 + disease with leptomeningeal carcinomatosis were treated with trastuzumab before or during the LMC diagnosis. Among the 26 patients with brain metastasis and HER2 + disease, five did not receive any treatment (three declined systemic treatment, and two died before initiating any treatment). Of the remaining 21 patients, 16 received trastuzumab either before or after the diagnosis of brain metastasis, while five had an unknown systemic treatment history as they sought treatment at our center specifically for brain radiation.

### Time to develop brain metastasis

The median time to develop CNS metastases, calculated from the primary cancer diagnosis to CNS metastasis, was different between patients with BrM HER2 positive, BrM HER2 negative and leptomeningeal carcinomatosis. Patients with BrM and HER2-positive disease developed BrM late into their cancer course, with a median of 12 months (95% CI, 9.7–19.1 months). Alternatively, patients with BrM and HER2-negative disease and patient with leptomeningeal carcinomatoses, developed CNS metastasis with a median of 6.7 (95%, CI 3.4,13.8) and 4.9 months, respectively (Table 3).

**Table 3:** Time to develop brain metastases and leptomeningeal carcinomatosis*

Time do develop CNS metastasis	Median in Months (95% CI)
Brain Metastases (HER2 positive)	12.0 (9.7, 19.1)
Brain Metastases (HER2 negative)	6.7 (3.4, 13.8)
Leptomeningeal Carcinomatosis	4.9 (2.0, 8.4)

*From diagnosis of gastroesophageal carcinoma until date of CNS diagnosis

### Number and distribution of CNS metastases and survival

**Fig. 4:**
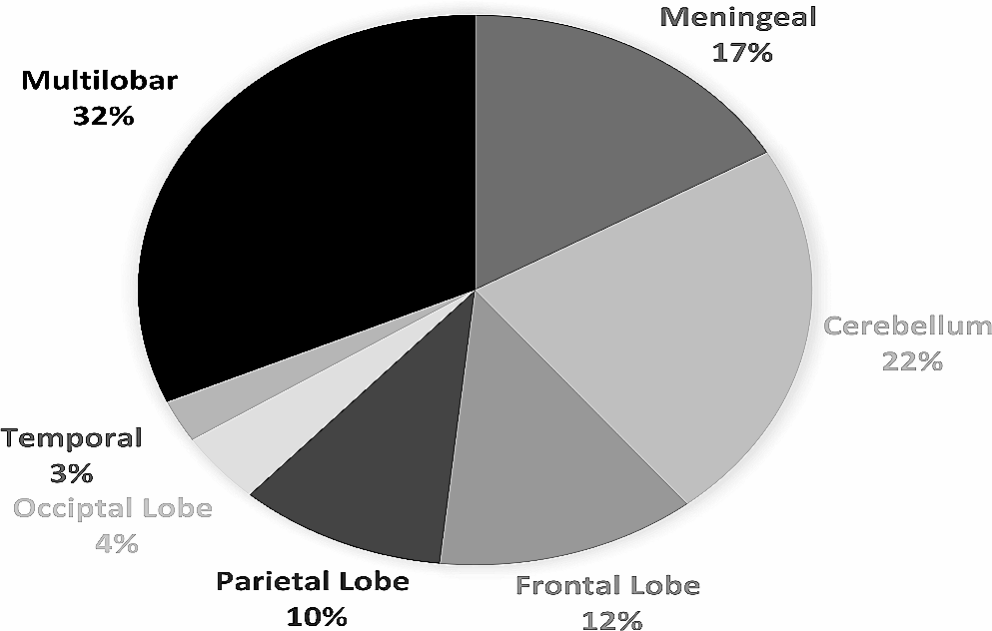
Baseline distribution of CNS metastasis location

Most GEC patients with CNS metastases [100 (82%)] were brain metastases, while only [20 (16,6%)] had leptomeningeal carcinomatosis. Among those with brain metastases, multilobe disease was prevalent in 32% of cases. The most common locations for BrM were the cerebellum (27 [22%]), followed by the frontal lobe (14 [12%]) (Fig. 4).

### Survival analysis

**Fig. 5:**
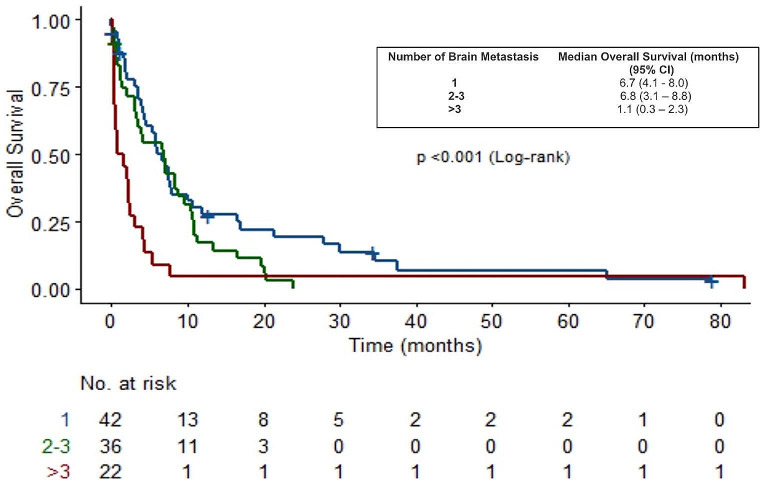
OS in patients with 1; 2–3 and > 3 Brain metastases

The median OS for patients with one single metastasis was 6.7 months (95% CI, 4.1-8.0) and those with 2–3 metastases had a median OS of 6.8 (95% CI, 3.1–8.8), months. In contrast, patients with more than three metastases had a significantly lower median OS of 1.1 months (95% CI, 0.4–2.3), (*p*-value < 0.001) (Fig. 5).

**Fig. 6:**
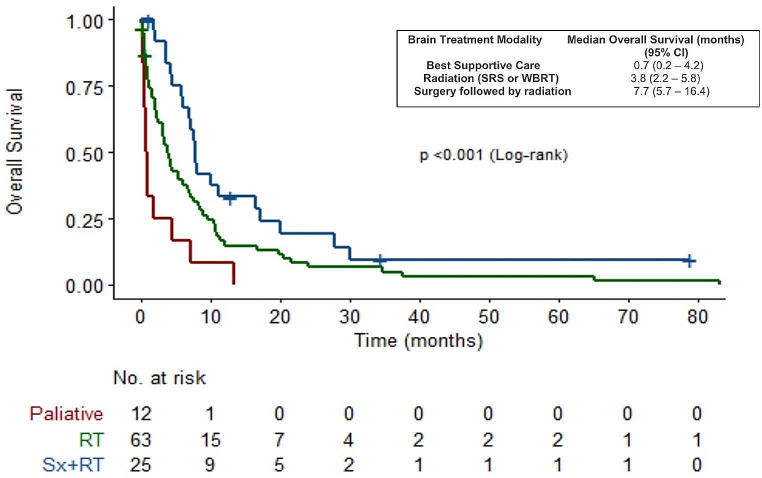
OS and treatment modalities in patients with Brain metastasis

In the group of patients with brain metastases, the median survival varied depending on the treatment approach. Those who received best supportive care had a median survival of 0.7 months (95% CI, 0.2–4.2), while patients treated with radiation alone had a longer median survival of 3.8 (95% CI, 2.2–6.6) months. Notably, patients who underwent surgery followed by radiation had the most extended median survival of 7.7 (95% CI, 5.7–16.5) months (*p* < 0.001) as illustrated in Fig. 6.

**Fig. 7:**
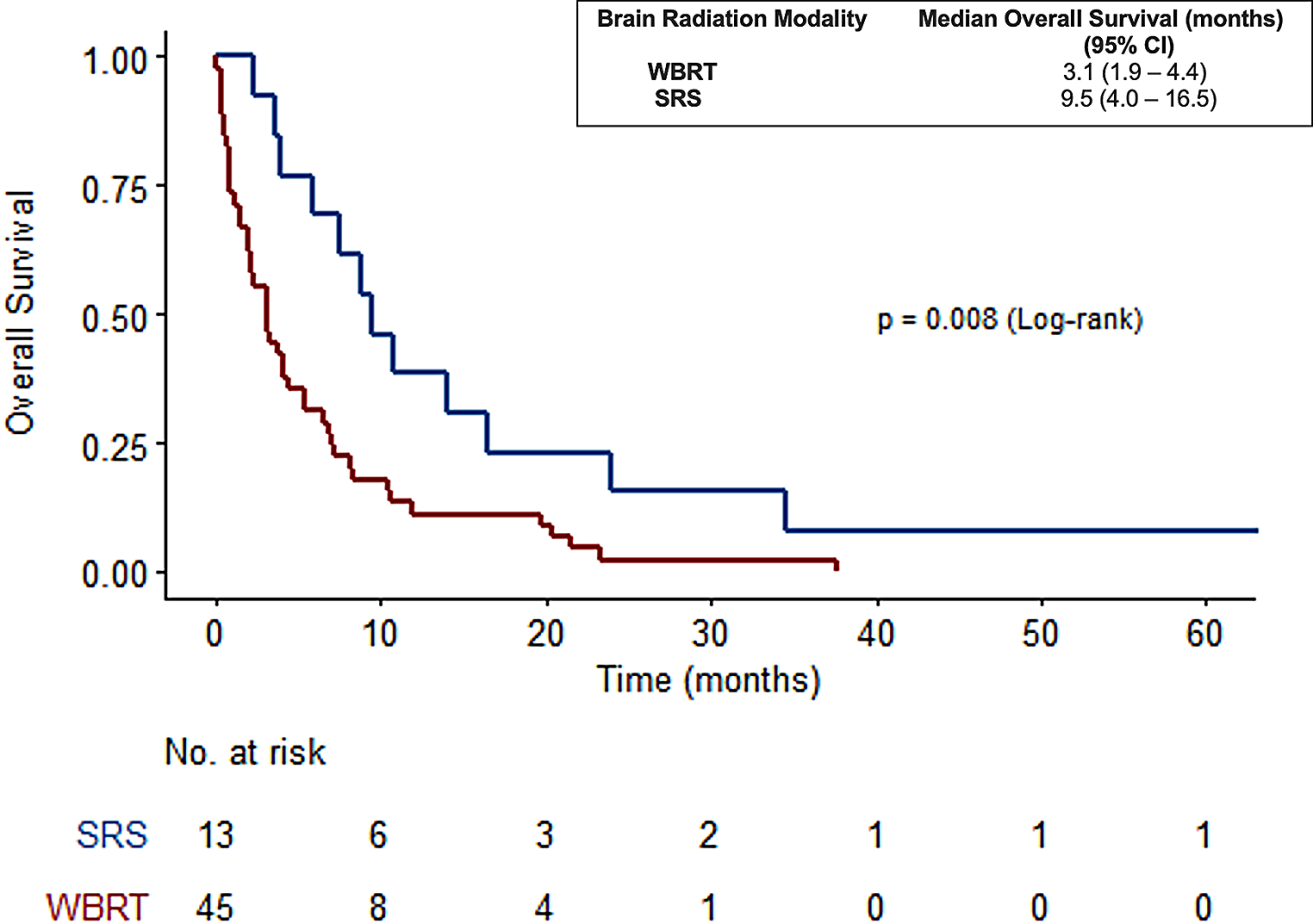
OS in WBRT and SRS group in patients with metastasis

The overall survival rates in the group of patients with brain metastasis treated with radiation was 3.8 months. The difference in survival was calculated between the two radiation modalities (SRS and WBRT). The median survival time was 9.53 months (95% CI: 4.0–16.5.) in the SRS group and 3.13 months (95% CI: 1.9–4.4) in the WBRT with a statistically significant difference in OS between the two groups (*p* = 0.008) as seen in Fig. 7. Patients treated with SRS had a higher functional status (100% had ECOG 0 or 1) and out of the patient cohort, 6 out of 13 individuals had HER2 positive disease and underwent systemic treatment either right before or after receiving brain radiation. The patients who received SRS generally had a lower count of brain metastases, as 100% (13/13) of them presented with only 1 to 3 brain metastases (Table 4).

**Table 4:** Patients with brain metastasis treated with radiation

Radiation Modality	Number of Patients	Number of Brain Metastasis	HER2 status *N* (%)
WBRT	45	**1–3**: 30; ≥4: 15	13 (28%)
SRS	13	**1–3**: 13; ≥4: 0	6 (46%)
Unknown	5		

The multivariate analysis showed a higher probability of death in brain metastasis patients with ECOG performance status ≥ 2 *(HR, 2.6; 95% CI, 1.4, 4.8; p 0.002)*, number of BrM ≥ 4 *(HR, 2.8; 95% CI, 1.5, 5.1;* *p* < 0.001), number of metastatic sites (HR, 1.2; 95% CI, 1.1, 1.5, p 0.009) and predicted superior survival in patients who received surgery followed by radiation *(HR,0.4; 95% CI, 0.1, 0.9; p 0.03).* (Table 5.)

**Table 5:** Multivariable Cox Model (only patients with brain metastasis)

Variable	Hazard Ratio (95% CI)	*p*-value
**Age**	1.00 (0.978, 1.025)	0.89
**Number of Brain Metastasis**		**0.004**
1	Reference	
2–3	1.5 (0.9, 2.6)	0.12
≥ 4	2.8 (1.5, 5.1)	**< 0.001**
**Number of Metastatic sites**	1.3 (1.1, 1.5)	**0.009**
**Treatment Modality**		
Palliative care only/ Best Supportive Care	Reference	
Radiation Only (SRS/WBRT)	0.4(0.2, 0.9)	0.02
Surgery Followed by Radiation	0.4(0.1, 0.9)	**0.03**
**ECOG (CNS presentation)**		**0.002**
0–1	Reference	
≥2	2.6 (1.4, 4.8)	

Abbreviations: ECOG – Eastern Cooperative Oncology Group (ECOG) SRS - stereotactic radiosurgery, WBRT – Whole Brain Radiation Therapy, NA not Applicable

**Fig. 8:**
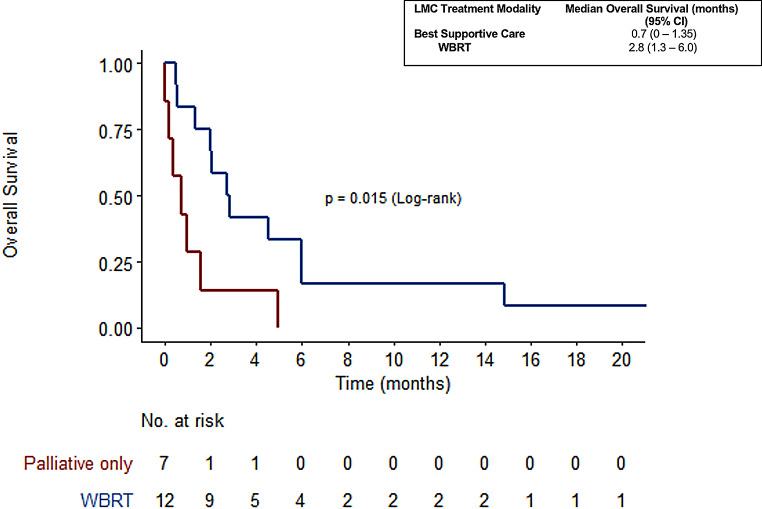
OS and treatment modalities in patients with Leptomeningeal Carcinomatosis

In patients with leptomeningeal carcinomatosis, the median survival also differed based on the treatment received. Patients who received BSC had a median survival of 0.7 months (7 out of 20 patients), whereas those who underwent WBRT had a significantly longer median survival of 2.8 months (12 out of 20 patients) (*p* = 0.015) shown in Fig. 8.

## Discussion

Brain metastasis is a rare complication of esophageal and gastric cancer, with reported incidences in the literature ranging from 0.7 to 6.6%. Leptomeningeal carcinomatosis is even rarer, estimated to occur in 0.16–0.19% of gastric cancers [10]. In our database of 3,283 patients, we observed an incidence of 3.04% for BrM and 0.61% for LMC. Notably, some patients diagnosed with GEC were not consistently treated or followed up in our institution, introducing variations in reported CNS metastasis frequencies. Upon focusing on a subgroup of 2,230 patients actively monitored for at least 5 years or until death, recalculated frequencies for BrM and LMC were 4.48% and 0.89%, respectively. The frequency of BrM aligns with previous literature, while leptomeningeal carcinomatosis showed a slightly higher incidence.

All leptomeningeal carcinomatosis cases in our study were associated with adenocarcinoma histology. However, in patients with brain metastasis, approximately 15% had squamous cell histology. Brain metastasis is exceptionally uncommon in esophageal squamous cell carcinoma (ESCC), constituting merely 0.3% of cases according to a study involving 4494 diagnosed patients between 2010 and 2015 [20]. Conversely, a study from M. D. Anderson Cancer Center revealed that among 1085 patients with esophageal adenocarcinoma, 2.0% experienced brain metastasis, while only 0.4% of the 405 patients with ESCC demonstrated brain metastasis [21], compatible with the incidence of ESCC patients in our study, 0.42%.

Genomic analyses comparing brain metastases to their primary tumors and other extracranial metastases have unveiled the presence of potentially actionable driver mutations unique to brain metastases [22]. In HER2 positive breast cancer (BC), there exists a notably elevated incidence of brain metastases when compared to other subtypes, suggesting a distinct affinity of HER2 positive cancer cells for the central nervous system [23]. The documented incidence of patients with gastroesophageal adenocarcinoma metastatic to the brain and HER2 positive disease spans a range from 37.3% to 85,7% [6, 24]. Consistent with previous research [12–14], our study also observed an enrichment of HER2 expression in gastroesophageal adenocarcinoma patients with brain metastases with a frequency of 60% in patients with known HER2 status. This finding was uncommon in patient with leptomeningeal carcinomatosis, with only 15% harboring HER2 disease.

In previous studies involving individuals with brain metastasis have not demonstrated a significant discrepancy in the time to develop brain metastasis between those with HER2-positive and HER2-negative statuses [25]. However, in our research, patients with HER2-positive disease showed a delayed time to develop brain metastasis, with a median of 12 months (95% CI, 9.7–19.1 months), as opposed to 6.7 months (95% CI, 3.4–13.8) for their HER2-negative counterparts. Most of patients with HER2-positive disease received anti-HER2 treatment which might have contributed for this outcome. Conversely, individuals with leptomeningeal carcinomatosis displayed a faster onset of CNS metastasis, with a median time to leptomeningeal carcinomatosis of merely 4.9 months, occurring at an early stage of metastatic disease. Molecular analyses showed a higher incidence of diffuse type carcinoma among patients with leptomeningeal carcinomatosis (85%), aligning with established literature characterizing this subtype of gastric cancer as notably aggressive [26–28].

In the context of a rare tumor, where evidence regarding its potential impact on mortality is lacking, effective treatments for brain involvement are limited, and information on cost-effectiveness is scarce, the National Comprehensive Cancer Network (NCCN) do not recommend routine brain MRI staging for the gastroesophageal population [29]. Nonetheless, the NCCN recommends brain MRI when patients present with neurological symptoms. Brain metastases can present in various ways, mainly linked to the expansion of the tumor mass and the resulting edema, causing symptoms in most patients [30]. In our study, the most prevalent symptoms were sensory or motor neurological deficits, followed by headaches and confusion, encompassing both brain metastasis and leptomeningeal patients. Additional symptoms included ataxia, seizures, nausea, vomiting, and dizziness. When these symptoms are present, further investigation for potential brain metastasis should be considered.

Considering the location of a brain metastasis is a fundamental aspect of clinical practice when determining appropriate local therapies [31]. Existing studies have predominantly either found no discernible impact on survival or indicated that brainstem and cerebellum lesions are associated with a poorer prognosis [31]. Among those with brain metastases, multilobe disease was prevalent in 32% of cases. The cerebellum emerged as the most frequent site for brain metastases (22%), corresponding with challenging locations noted in the literature and reflecting its prognostic significance.

Numerous validated scoring systems and factors exist for prognosticating brain metastases across various cancer types [32–34]. The significant prognostic elements, however, exhibit variability depending on the specific diagnosis. For instance, in the context of lung cancer, these factors encompass the Karnofsky Performance Status (KPS), age, presence of extracranial metastases, and the number of brain metastases, aligning with the original Lung-GPA scoring system. On the other hand, for melanoma and renal cell cancer, prognostic factors primarily involve the KPS and the number of brain metastases [34]. In our study, we observed that factors such as the presence of extracranial sites, an ECOG performance status of ≥ 2, and having four or more brain metastases were associated with reduced survival rates.

Local therapies for brain metastases include surgery, SRS, WBRT, or some combination of these [35, 36]. Within the scope of our study, the most favorable outcomes were observed when surgery was followed by radiotherapy, resulting in a median OS of 7.7 months. Historically, WBRT was widely in brain metastases cases non candidates for surgery. However, recent advancements in SRS have revolutionized treatment possibilities by enabling targeted delivery of higher radiation doses to specific disease areas, mitigating the substantial adverse effects linked with WBRT, particularly cognitive impairment [37–39]. Consequently, SRS has taken precedence over WBRT as the favored radiation therapy modality, reserving WBRT for cases of widespread disease. The median survival for patients who exclusively received radiation therapy for brain metastases, whether through WBRT or SRS, was 3.8 months. A comparative analysis between patients who underwent WBRT and SRS was conducted. Notably, patients who received SRS exhibited a significantly extended survival, in contrast to those who received WBRT. It is important to acknowledge that nearly 10% of the patient population lacked information in their medical records regarding the specific treatment modality they received. It is likely that this benefit may be influenced by a selection bias in the choice of treatment modality since patients treated with SRS had a higher functional status and almost 50% of individuals had HER2 positive disease and underwent systemic treatment either right before or after receiving brain radiation. This could potentially be a significant factor contributing to improved survival outcomes in patients who received SRS. Additionally, it’s worth noting that the patients who received SRS generally had only 1 to 3 brain metastases, also favoring a better outcome in this population.

The survival outcomes in patients with leptomeningeal carcinomatosis is less than 3 months in patients with gastric cancer, previously described in case reports [40]. We were able to show in this analysis the survival in leptomeningeal carcinomatosis and its treatment modalities. Patients who received BSC had a median survival of 0.7 months, whereas those who underwent whole brain radiation therapy (WBRT) had a significantly longer median survival of 2.8 months with statistical significance.

The treatment scheme in gastric cancer patients harboring CNS metastasis should be individualized and based on expected survival, performance status, symptoms, the number, location, and size of metastases [35, 36].

### Potential limitations

There are notable constraints regarding the accurate incidence and outcomes of patients CNS metastases due to the rarity of this tumor, especially the data coming from single center institution.

Furthermore, our study had several notable limitations worth highlighting. It’s crucial to acknowledge that our research was carried out retrospectively, potentially resulting in incomplete or unavailable data. For example, HER2 status was unknown (not reported) for 53% of patients with gastroesophageal adenocarcinoma.

Given the wide array of systemic treatments administered and their varying timing concerning the onset of brain metastases, we encountered challenges in assessing any potential correlation between systemic therapy and the survival of patients with BrM.

Lastly, it’s important to consider that patients who underwent surgery followed by radiation for brain metastasis, or those who received WBRT for leptomeningeal carcinomatosis, exhibited improved outcomes. These findings could potentially be attributed to a selection bias in opting for these treatment modalities, potentially involving factors like a lower number of brain metastases and a higher overall performance status.

## Conclusion

In our retrospective cohort study, we observed a higher prevalence of HER2 positivity in cases of brain metastasis compared to leptomeningeal carcinomatosis. Notably, patients with brain metastasis and HER2-positive disease exhibited a tendency to manifest brain metastasis at a later stage compared to those with HER2-negative disease. Additionally, our findings indicated that among patients with brain metastases from gastroesophageal diseased patients who underwent brain metastasis resection followed by radiotherapy had an improved survival. Furthermore, individuals with leptomeningeal carcinomatosis who underwent whole-brain radiotherapy exhibited prolonged survival compared to those receiving best supportive care. Nevertheless, it is essential to recognize the potential impact of selection bias, including factors such as a lower number of brain metastases and a higher overall performance status, on the selection of treatment modalities. To address these questions with greater precision, multicenter studies, prospective investigations, and clinical trials would be instrumental.

## Data Availability

No datasets were generated or analysed during the current study.
